# Spatial Scales of Bacterial Diversity in Cold-Water Coral Reef Ecosystems

**DOI:** 10.1371/journal.pone.0032093

**Published:** 2012-03-05

**Authors:** Sandra Schöttner, Christian Wild, Friederike Hoffmann, Antje Boetius, Alban Ramette

**Affiliations:** 1 HGF-MPG Joint Research Group on Deep-Sea Ecology and Technology, Max Planck Institute for Marine Microbiology, Bremen, Germany; 2 Coral Reef Ecology Group, Leibniz Center for Tropical Marine Ecology, Bremen, Germany; 3 Center for Geobiology, University of Bergen, Bergen, Norway; 4 Uni Environment, Uni Research AS, Bergen, Norway; Heriot-Watt University, United Kingdom

## Abstract

**Background:**

Cold-water coral reef ecosystems are recognized as biodiversity hotspots in the deep sea, but insights into their associated bacterial communities are still limited. Deciphering principle patterns of bacterial community variation over multiple spatial scales may however prove critical for a better understanding of factors contributing to cold-water coral reef stability and functioning.

**Methodology/Principal Findings:**

Bacterial community structure, as determined by Automated Ribosomal Intergenic Spacer Analysis (ARISA), was investigated with respect to (i) microbial habitat type and (ii) coral species and color, as well as the three spatial components (iii) geomorphologic reef zoning, (iv) reef boundary, and (v) reef location. Communities revealed fundamental differences between coral-generated (branch surface, mucus) and ambient microbial habitats (seawater, sediments). This habitat specificity appeared pivotal for determining bacterial community shifts over all other study levels investigated. Coral-derived surfaces showed species-specific patterns, differing significantly between *Lophelia pertusa* and *Madrepora oculata*, but not between *L. pertusa* color types. Within the reef center, no community distinction corresponded to geomorphologic reef zoning for both coral-generated and ambient microbial habitats. Beyond the reef center, however, bacterial communities varied considerably from local to regional scales, with marked shifts toward the reef periphery as well as between different in- and offshore reef sites, suggesting significant biogeographic imprinting but weak microbe-host specificity.

**Conclusions/Significance:**

This study presents the first multi-scale survey of bacterial diversity in cold-water coral reefs, spanning a total of five observational levels including three spatial scales. It demonstrates that bacterial communities in cold-water coral reefs are structured by multiple factors acting at different spatial scales, which has fundamental implications for the monitoring of microbial diversity and function in those ecosystems.

## Introduction

Cold-water coral (CWC) reef ecosystems are increasingly portrayed as biodiversity hotspots on continental margins, seamounts and mid-ocean ridges around the world [1]. They appear as speciose, abundant and widespread as their warm-water counterparts [2]–[5], and represent important species pools [6]–[8] and speciation centers [9] in the deep sea. Their potential to foster a high degree of local diversity and biomass is assumed to be rooted in the ecosystem engineering capacity of scleractinian corals [10], [11], such as the cosmopolitan key species *Lophelia pertusa* (L. 1758, Caryophylliidae) and *Madrepora oculata* (L. 1758, Oculinidae). By forming enormous dendritic skeletal frameworks, these corals provide complex three-dimensional living space for a plethora of mobile and sessile organisms [7], [8], [12]. They also alter flow regimes and sedimentation rates, thereby modifying the abiotic environment in time and space ([1] and references therein).

Often, structural complexity in CWC reefs is promoted by pronounced ecosystem heterogeneity. Unlike warm-water coral ecosystems that constitute relatively contiguous reef environments with clear wave and sun energy-related zoning [13], CWC ecosystems can consist of isolated colonies, small patch accumulations, large reefs, or giant carbonate mounds, and differ substantially with respect to their spatial configuration [14]–[19]. Often, individual clusters of coral frameworks form entire reef complexes which, depending on local seabed geology as well as community history (i.e. the combined effects of past community assembly, succession and interaction, including individual life trajectories and trade-offs), exhibit distinctive geomorphologic and taphonomic (i.e. seabed form- and fossilization-related) zoning, with marked transitions in sediment type, faunal composition and proliferation stage [7], [19]–[21].

Despite mounting evidence of warm-water coral reefs as structured landscapes of complex microbial communities [22]–[24], insights into the microbial diversity of CWC ecosystems are limited. Deciphering principle patterns of microbial community variation may, however, prove critical for a better understanding of factors contributing to CWC reef stability and functioning. Especially bacteria play important ecological roles for corals and entire reef systems by contributing substantially to biogeochemical processes, invertebrate life cycles, host metabolism, protection and adaptation, as well as to overall species diversity (e.g. [24] and references therein). Hence, their spatial and temporal dynamics are relevant features for the functioning of coral ecosystems.

So far, studies of CWC reef microbiology mainly focused on the identification of bacteria associated with scleractinians [25]–[32] or octocorals [33]–[37]. Community fingerprinting methods (e.g. Automated Ribosomal Intergenic Spacer Analysis, ARISA; Terminal Restriction Fragment Length Polymorphism, T-RFLP; Denaturing Gradient Gel Electrophoresis, DGGE) and 16 S rRNA gene sequencing were used to show that bacterial assemblages colonizing living scleractinians and octocorals differ from those of dead corals or from those of the ambient environment like seawater or sediments [25], [26], [28], [29], [32], [33]. Even distinct coral-generated microbial habitats, such as branch surface, mucus and tissue, were found to exhibit specific bacterial community signatures [26], [29], [32]. Spatial patterns of bacterial communities associated with different octocoral species appeared either highly distinct [37] or conserved between different reef sites (and across an environmental impact gradient [36]).

On *L. pertusa*, several bacterial sequences were shared across geographically separate regions, such as the Gulf of Mexico and the Trondheimsfjord in Norway [28], [30]. Strict host-specificity of *L.pertusa*-associated bacteria was so far not evidenced due to significant community variations between (i) sampling locations within the same geographic area or reef complex [26], [28], [29], [30], (ii) colonies of the same coral species [29], (iii) single polyps within the same coral colony [29], and (iv) differently colored types within the same coral species [28]. In fact, current evidence suggests that coral-bacteria associations considerably differ with both coral-derived microbial habitats and prevailing environmental conditions. However, due to the variations in spatial scale and methodology applied in aforementioned surveys, the relative importance of spatial and reef-organizational factors that determine bacterial biogeography across various scales is not evident.

**Figure 1:**
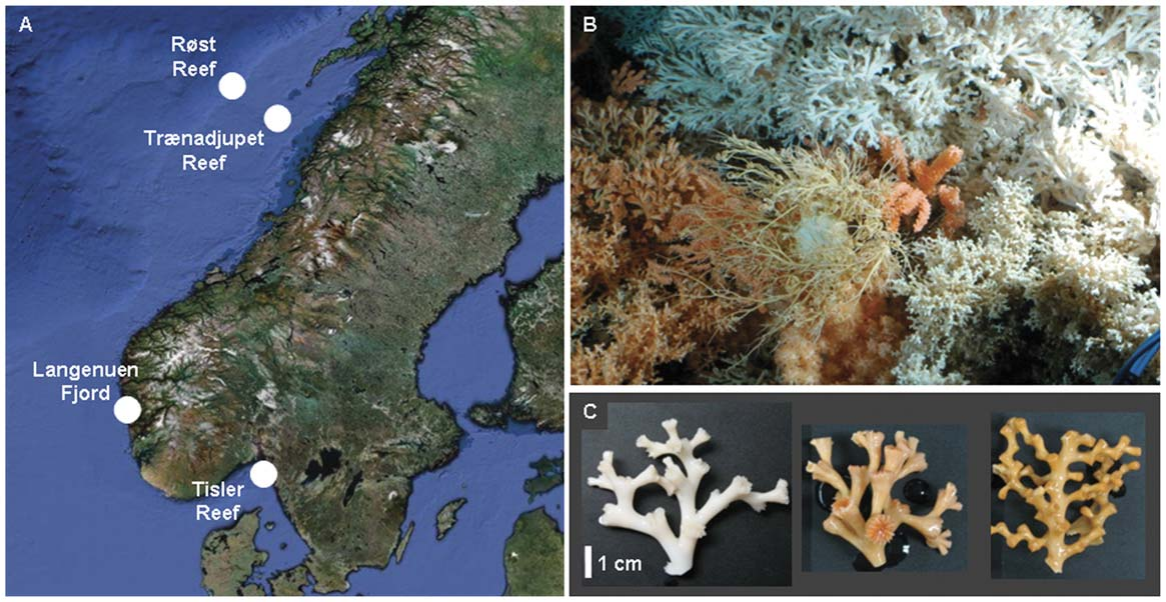
Reef sites and corals targeted in this study. (**A**) Offshore and CWC ecosystems along the Norwegian continental margin. (**B**) Living colonies of *Lophelia pertusa* and *Madrepora oculata* in their natural environment at Røst, northern mid-Norwegian continental margin. (**C**) Fragments of freshly sampled white *L. pertusa* (*left*), red *L. pertusa* (*middle*), and red *M. oculata* (*right*).

**Figure 2:**
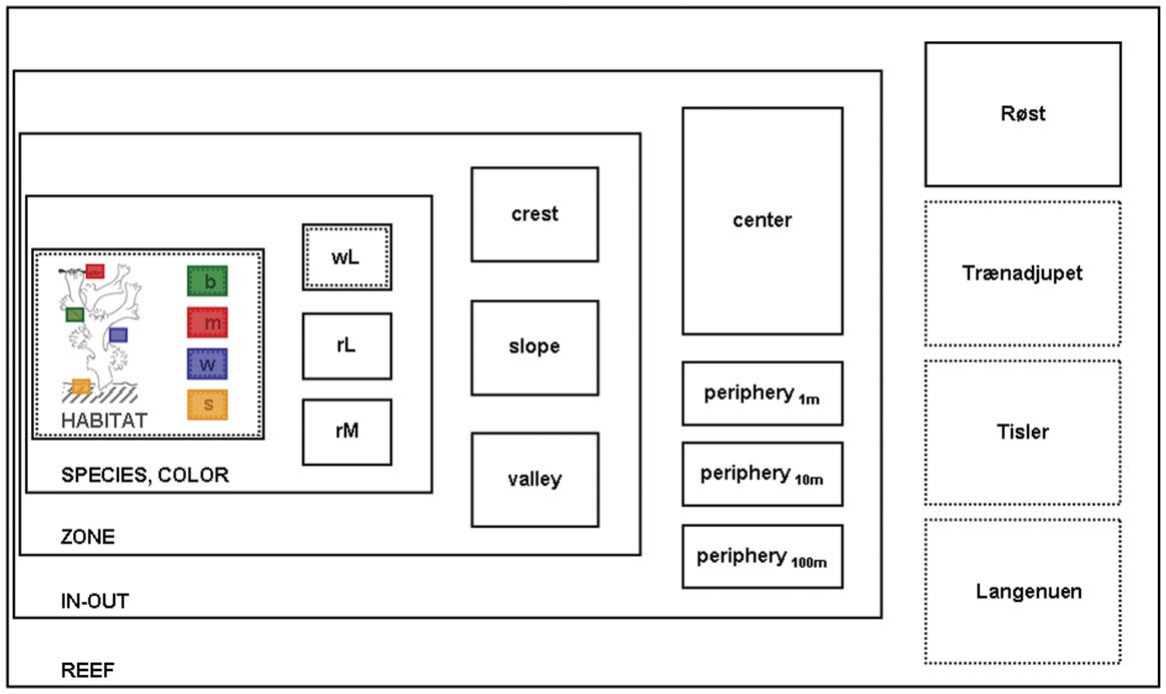
Multi-scale, hierarchical sampling design. Nested frames indicate the different levels of observation, increasing in scale from inside (*left*) to outside (*right*). Boxes within frames symbolize each lower level as integral part of respective next higher level. At the main study site, Røst, sampling was implemented on all levels (continuous line); at all other sites, it was performed only on the lowest and highest level, respectively (dotted line). The following levels and scales of observation were considered: 1) HABITAT (µm–cm): coral branch surface (“b”), coral mucus (“m”), ambient seawater (“w”), proximal sediments (“s”); 2) SPECIES (cm–m) – white *Lophelia pertusa* (“wL”), red *Lophelia pertusa* (“rL”), red *Madrepora oculata* (“rM”); 3) ZONE (1 m–10 m): ridge top with coral terraces (“crest”), ridge slope with single coral colonies on rubble (“slope”), ridge depression with single colonies on clay (“valley”); 4) IN-OUT (1 m–10 m–100 m): reef center (“reef-in”), reef periphery in distances of 1, 10, 100 m away from the apparent reef margin (“reef-out”); 5) REEF (km): Røst Reef (“Røst”), Trænadjupet Reef (“Trænadjupet”), Tisler Reef (“Tisler”), Langenuen Fjord (“Langenuen”).

The aim of the present study was to identify patterns of bacterial communities in CWC reefs (Fig. 1) using a multi-scale, hierarchical sampling approach spanning five study levels including three spatial scales (Fig. 2). Sources of bacterial community variation were assessed from local (intra-reef) to regional (inter-reef) scale by considering (i) microbial habitat type on and around coral colonies (coral branch surface, coral mucus, ambient seawater, proximal sediments), (ii) coral species (*L. pertusa*, *M. oculata*) and color phenotype (white, red), (iii) geomorphologic reef zoning (ridge crest, slope, depression), (iv) reef boundary (up-slope reef center, down-slope reef periphery), and (v) reef site (Røst Reef, Trænadjupet Reef, Tisler Reef, Langenuen Fjord) and proximity to shore (offshore, inshore).

For this purpose, bacterial community DNA derived from two constructional corals *L. pertusa* and *M. oculata*, as well as from associated seawater and surface sediments, was collected from four CWC reef ecosystems on the Norwegian continental margin (Fig. 1). The community fingerprinting approach ARISA then allowed for a time- and cost-effective analysis of the large, heterogeneous sample set. Despite the lack of information on OTU identity, ARISA was chosen for its proven ability to provide robust insights about bacterial community dynamics at different spatial (and temporal) scales (e.g. [38], [39]). Ultimately, the different but not mutually exclusive sources of bacterial community variation were disentangled by quantifying their respective effects with multivariate statistics.

## Results

### Variation in bacterial OTU number and occurrence

**Figure 3:**
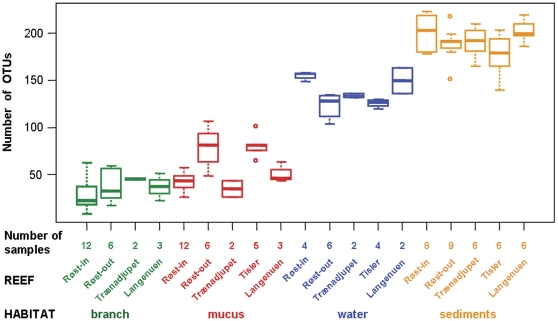
Number of ARISA-derived OTUs in distinct microbial habitats at each reef site. Top, middle, and bottom lines of the boxes represent the 25th, 50th (median), and 75th percentiles, respectively, while the end of the whiskers represent the 5th and 95th percentiles, respectively; box height and symmetry around the median indicate the degree of dispersion and skewness in the data, respectively; outliers above and below the whiskers denote extreme values.

From a pool of 440 different operational taxonomic units (OTUs) occurring in the whole data set (104 samples), between 9–223 OTUs were obtained per sample. OTU number was strongly related to microbial habitat type (KW, P<0.001), and to the reef site (P = 0.05; Fig. 3). The most pronounced difference occurred between coral-generated surfaces and the ambient environment, with branch (34±15 OTUs) and mucus (58±22 OTUs) featuring 30–80% lower mean OTU numbers than water (135±16 OTUs) and sediments (192±19 OTUs). At Røst, mean OTU numbers showed a slight increase (P = 0.0398; Fig. 3) between reef center (Røst-in, 86±11 OTUs) and reef periphery (Røst-out, 116±62 OTUs), which was mainly related to branch and mucus variability. When studying local trends at Røst-in and Røst-out separately, however, neither geomorphologic reef zoning (P = 0.098), nor gradual distance away from the apparent reef margin (P = 0.956) resulted in any significant variation of OTU numbers. These were also not significantly related to coral species (*L. pertusa* and *M. oculata* with 36±13 and 35±17 OTUs, respectively; P>0.05) or to coral color (white and red colonies with 34±14 and 38±14 OTUs, respectively; P>0.05).

OTU partitioning among the four different microbial habitats showed that, from a total of 380–390 different OTUs detected in coral- water-, and sediment samples at Røst-in, only very few were exclusively present on *L. pertusa* and *M. oculata* surfaces of a given color phenotype (branch: <1–2%, mucus: 1%; Fig. S2A). Even with all coral samples from Røst-in combined, branch and mucus each contained only about 2% unique OTUs, from a total of 396 different OTUs (Fig. S2B). By contrast, the surrounding water and proximal sediments at Røst-in contained 4–5% and 19–28% unique OTUs, respectively, and therefore more of the microbial habitat-specific bacterial signatures (Fig. S2B). These patterns were confirmed for all study sites, with only minor variations (data not shown). OTU partitioning with the whole data set, i.e. with altogether 440 different OTUs from Røst, Trænadjupet, Tisler, and Langenuen combined (Fig. S2B), revealed clearly lower fractions of water- and sediment-specific OTUs (<1% and 5%, respectively), while those of branch- and mucus-specific OTUs remained virtually unchanged (branch: 2–3%, mucus: 1–2%). Concomitantly, the fraction of shared OTUs increased considerably from local (Røst-in: 4–55 and 9%, Fig. S2A) to regional scale (all sites: 27%; Fig. S2B), indicating a decrease in habitat-specificity for water- and sediment-associated OTUs, due to their partial presence in branch and mucus samples from other sites.

**Figure 4:**
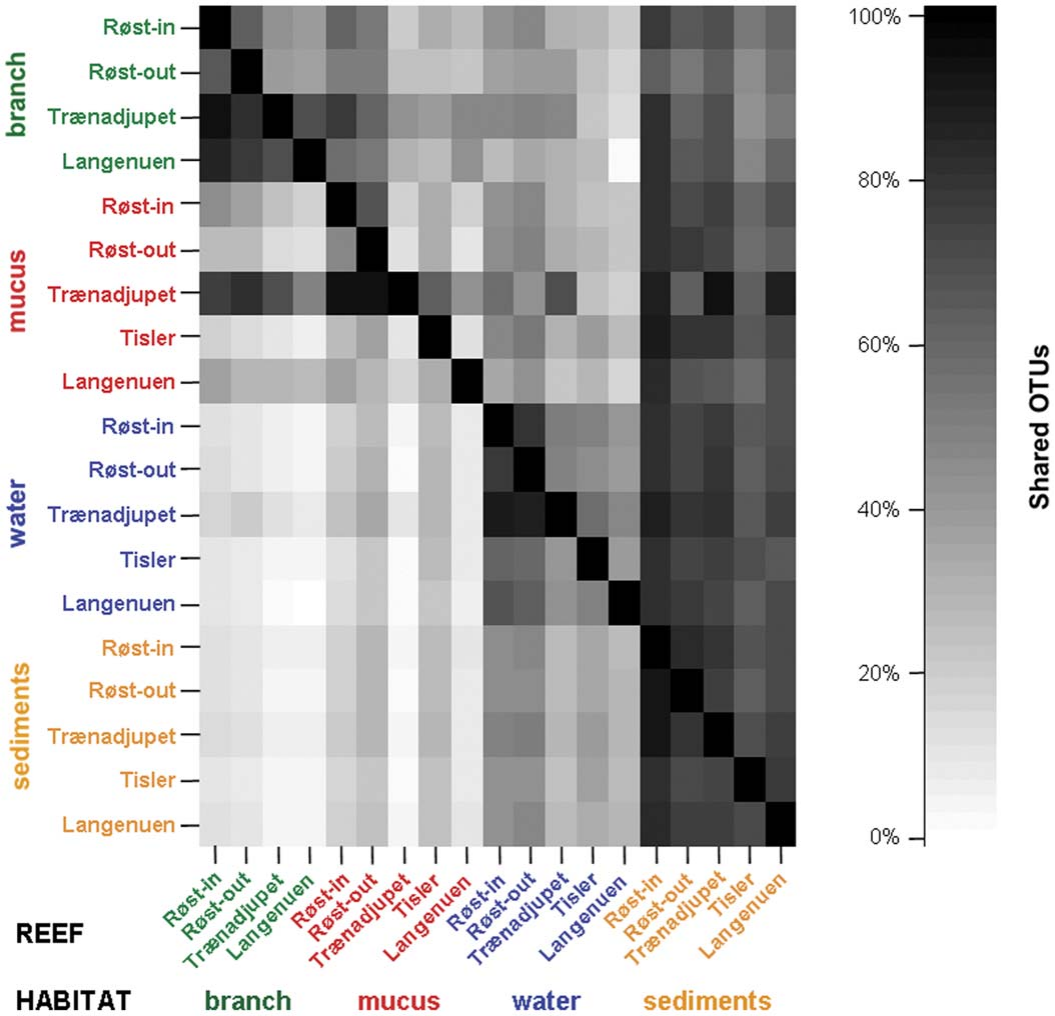
Pairwise comparison of OTU overlap between microbial habitats at each reef site. Samples were grouped according to microbial habitat type and reef site. In this asymmetrical representation, rows correspond to the reference group and columns to the group being compared. It provides an overview of potential directional dynamics between different microbial habitat types, with the respective fraction (%) of shared OTUs indicated by different degrees of shading.

The detailed analyses of OTU overlap between samples confirmed those habitat-specific trends and distinguished particularly coral-derived surfaces from the surrounding environment (Fig. 4). Differences in OTU overlap clearly reflected variations in OTU number, with OTU-poor habitats (branch, mucus) sharing a much higher percentage of their OTU pool with OTU-rich habitats (water, sediments) than reciprocally. Branch and mucus shared at least half of their OTUs with sediments (50% and 73%, respectively), and a comparatively lower fraction with water (25% and 36%, respectively). Conversely, only 9–10% and 17–18% of all water and sediment OTU were found among branch and mucus OTUs, respectively. The number of OTUs shared solely between both coral-associated habitats amounted to a third of their respective OTU content (33–34%), whereas the water shared a much higher fraction of its OTU pool with the sediments (74%) than vice versa (34%). Between different reef sites (within each habitat separately; Fig. 4), the mean number of OTUs overlapping between any two reefs was the lowest in mucus (mean: 37%) and also the most variable (range: 11–94%). In contrast, reef-specific OTU fractions were usually the highest in sediments (76%), with relatively high and constant OTU overlap among reefs (63–90%). Overall, no difference between offshore (Røst, Trænadjupet) and inshore (Tisler, Langenuen) reef sites was detected (Fig. 4).

### Differences in community structure

**Figure 5:**
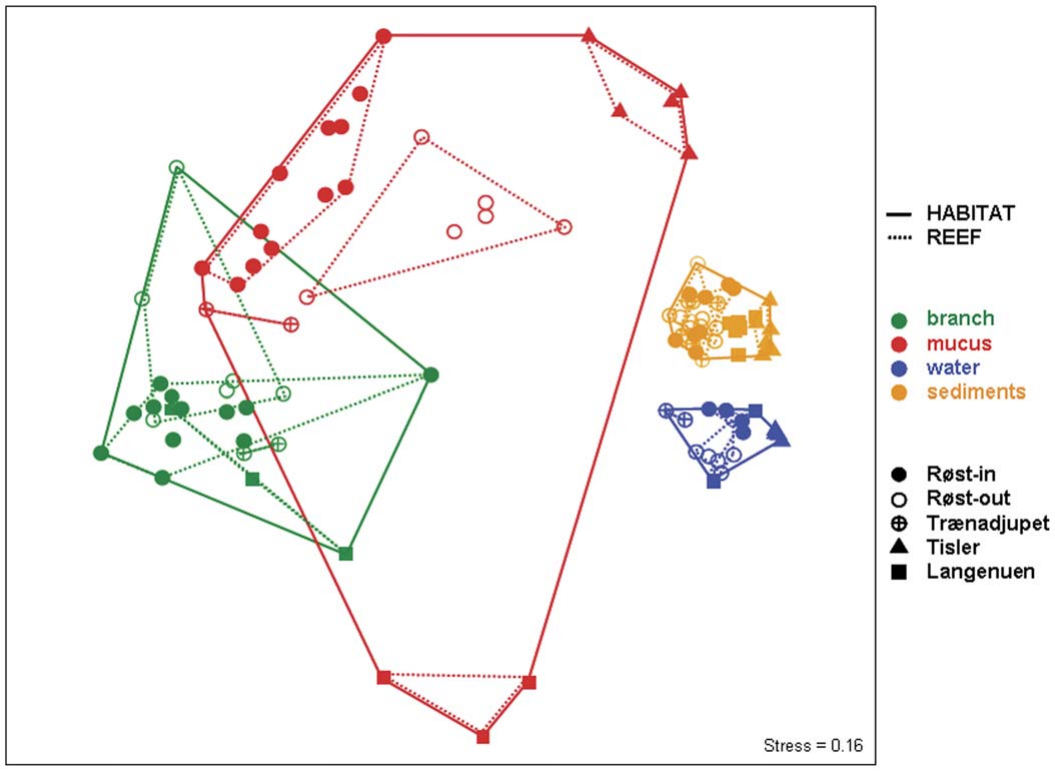
NMDS ordination of all ARISA community profiles. For each sample, consensus signals of PCR triplicates were used. Objects represent consensus signals for all PCR triplicates per sample and share a more similar community structure when plotting closer to each other (Bray-Curtis distance). *A posteriori* groupings specify microbial habitat type and reef site. The low stress value indicates appropriate representation of the original Bray-Curtis dissimilarity matrix into a 2-dimensional space.

Bacterial community structure strongly differed between microbial habitat types, with the greatest difference between coral-associated surfaces and ambient environment (Fig. 5, Fig. S3) as confirmed by PERMANOVA (Table 1). At Røst-in, as well as for all study sites combined, distinct community patterns were associated with branch, mucus, water or sediments. For the other reef sites (Table 1), the microbial habitat-specific separation was also similarly pronounced (PERMANOVA R^2^ = 0.75–0.80). In addition, bacterial community structure associated with each of the four reef systems clearly differed from each other, especially between offshore (Røst, Trænadjupet) and inshore (Tisler, Langenuen) sites (Table 1). These regional, inter-reef differences appeared particularly pronounced for mucus communities (Fig. 5, Fig. S4), but also for water and sediment communities.

**Table 1:** PERMANOVA of regional, inter-reef bacterial variation at *all study sites* as related to reef site (REEF) and microbial habitat type (HABITAT).

Test factor^a^	Test group	R^2^ b	F-ratio	P^c^
HABITAT	all samples	0.467	17.222	0.001 ***^d^
HABITAT	Røst	0.777	20.947	0.001 ***^d^
HABITAT	Trænadjupet	0.794	10.273	0.001 ***^d^
HABITAT	Tisler	0.801	24.135	0.001 ***^d^
HABITAT	Langenuen	0.748	9.888	0.001 ***^d^
REEF	all samples	0.124	2.795	0.001 ***^d^
REEF	branch	0.393	2.262	0.032 *
REEF	mucus	0.754	11.210	0.001 ***^d^
REEF	water	0.835	13.476	0.001 ***^d^
REEF	sediments	0.504	7.452	0.001 ***^d^

a Source of variation.

b Amount of explained variation.

c Significance level, assessed by 999 random permutations (*** P≤0.001, ** P≤0.01, * P≤0.05).

d Significance level below Bonferroni correction threshold.

At Røst, significant intra-reef differences were detected between the up-slope reef center (Røst-in) and the down-slope reef periphery (Røst-out) (Table 2). Similarly to the aforementioned regional patterns, this local pattern was mainly evidenced in mucus, water, marginally in sediments, but not in branch (Fig. S4). While those intra-reef differences in the Røst area indicated some degree of separation, the pronounced geomorphologic (including vertical) zoning at the reef center itself (Røst-in) revealed only a very weak trend among bacterial communities (Table 3, Fig. 3, Fig. S4). Communities did not change significantly over distances of 1, 10, and 100 m in the reef periphery (Røst-out; P>0.05).

**Table 2:** PERMANOVA of local, intra-reef bacterial variation at *Røst* as related to reef boundary, i.e. in-/out-reef location (IN-OUT).

Test factor^a^	Test group	R^2^ b	F-ratio	P^c^
IN-OUT	all Røst samples	0.048	3.100	0.006 **
IN-OUT	branch	0.095	1.684	0.073
IN-OUT	mucus	0.319	7.478	0.001 ***^d^
IN-OUT	water	0.300	3.433	0.01 *
IN-OUT	sediments	0.165	2.967	0.003 **

a Source of variation.

b Amount of explained variation.

c Significance level, assessed by 999 random permutations (*** P≤0.001, ** P≤0.01, * P≤0.05).

d Significance level below Bonferroni correction threshold.

**Table 3:** PERMANOVA of local, intra-reef bacterial variation at *Røst-in* as related to geomorphologic reef zoning (ZONE), coral species (SPECIES), coral color phenotype (COLOR), and microbial habitat type (HABITAT).

Test factor^a^	Test group	R^2^ b	F-ratio	P^c^
ZONE	all Røst-in samples			
ZONE	branch	0.143	0.752	0.752
ZONE	mucus	0.146	0.767	0.803
ZONE	water	0.399	1.326	0.260
ZONE	sediments	0.209	1.589	0.134
SPECIES^f^	all Røst-in samples	0.416	11.742	0.001 ***^d^
SPECIES^f^	branch	0.236	3.086	0.001 ***^d^
SPECIES^f^	mucus	0.163	1.942	0.043 *
COLOR^f^	all Røst-in samples	0.024	0.398	0.868
COLOR^f^	branch	0.011	0.878	0.607
COLOR^f^	mucus	0.082	0.631	0.794
HABITAT^f^	all Røst-in samples	0.587	23.411	0.001 ***^d^

a Source of variation.

b Amount of explained variation.

c Significance level, assessed by 999 random permutations (*** P≤0.001, ** P≤0.01, * P≤0.05).

d Significance level below Bonferroni correction threshold.

f Hierarchical sampling design, with each factor nested within the next higher one.

*L. pertusa* and *M. oculata* harbored significantly different bacterial assemblages (Table 3) despite some overlap (Fig. S4), and exhibited slight differences in their response to local reef complexity (Table S2A, C, B): While *M. oculata*-associated seemed to reflect changes in in-/out-reef location and geomorphologic reef zoning, *L. pertusa*-associated bacteria appeared more stable over space. In contrast, bacterial community variation related to coral color was, albeit overall significant, never supported, neither on branch nor in mucus (Table 3). All described results were also generally confirmed by ANOSIM and cluster analysis (data not shown).

## Discussion

### Microbial habitat type

Bacterial communities associated with the CWCs *L. pertusa* and *M. oculata* substantially differed according to the type of microbial habitat sampled. Coral branch surface, coral mucus, ambient seawater and proximal sediments each featured a specific community structure that significantly varied both in OTU composition and relative abundance. This habitat specificity seemed valid at all study sites, and confirmed earlier findings based on samples from one reef location [32]. Other studies have already reported evidence for bacterial habitat specificity in CWC reefs from the North-East Atlantic [26], [28], [29], the Central Mediterranean [25] and the Gulf of Alaska [33], or warm-water coral reefs (e.g. [40], [41]). The most pronounced differences concerned the distinction between bacterial communities associated with coral-generated surfaces and the ambient environment, in both OTU number and composition.

Not surprisingly, the difference in bacterial OTU number between the OTU-poor coral-associated and OTU-rich ambient microbial habitats strongly determined the overall degree of bacterial community partitioning and overlap: Irrespective of reef site and local zoning, branch and mucus exhibited notably few specific OTUs, as most of their respective OTU pool was shared with water and sediments. As expected, sediments generally exhibited the highest OTU abundance and number of specific OTUs, which was also previously observed in other ARISA-based studies on warm-water coral reefs (e.g. [38]).

Albeit strongly reduced in overall bacterial OTU number and specificity, bacterial communities on coral surfaces were characterized by high inter-sample, intra-habitat differences, clearly exceeding those of OTU-rich water and sediment communities. This may result from both stochastic events during community assembly, such as the random attachment of environmental bacteria on coral surfaces [42], and deterministic processes, such as the selection of few opportunists through the coral host (e.g. [43]), or antagonistic interactions between bacterial types [44]. Furthermore, this high variation in coral-associated assemblages may reflect local, inter-colony differences in host status, such as genetic identity [45], physiological condition [46], or developmental state ([47] and references therein). In their study of *M. oculata*-associated microbes, Hansson *et al.* [29] also reported significant inter-colony differences, which may even be further enhanced by intra-colony differences between single polyps [48], [49]. In addition to passive controls, bacterial colonization may also be actively regulated by the coral host (e.g. [50]) in adaptation to changing environmental conditions [51].

### Reef zoning, boundary and location

In general, bacterial communities mapped within the Røst reef center (Røst-in) revealed surprisingly similar patterns, despite the pronounced geomorphologic reef zoning (Table 3). This was unexpected, because seabed features often strongly affect and reflect local environmental dynamics of e.g. current regime, sediment deposition and diagenesis as well as organic matter quality, transport and remineralization within only few tens of meters (e.g. [18], [19]). Hence, the clearly distinguishable reef features present at Røst-in were assumed to significantly contribute to the structuring of bacterial assemblages, particularly so in water and sediments, but also in coral-derived microbial habitats.

The observed local similarity of bacterial community structure across reef-internal zones was not maintained beyond the reef center (Røst-in), due to significant community changes towards the reef periphery (Røst-out; Table 2). Only the branch communities remained similar, thereby marking an intriguing partition between the two coral-generated microbial habitats, branch and mucus. The scale-independent similarity of communities in branch *versus* mucus may be attributed to the circumstance that branch surface samples also included traces of coenosarc tissue, which may not only contain internal bacterial cells [31] but also exhibit external biofilm formation [43]. Those tissue- and biofilm-associated cells could be buffered more from exogenous change than mucus-inhabiting assemblages due to their embedding in the respective intra- and/or extra-cellular matrices, while the latter are more exposed to water column processes and thereby more prone to mirroring local, meso-scale spatial and environmental shifts. Within Røst, such shifts may occur as a function of the marked zone transition from the reef center to the periphery, with the latter representing an interface (“ecotone”; *sensu* [52]) between the structurally complex reef ecosystem and the more uniform, level bottom down-sloping into the abyssal plain [7]. Consequently, the underlying spatial and environmental changes that select for certain community structures may not necessarily follow linear distance relationships, but rather be subject to a whole interplay of locally different, ecosystem-specific factors.

The presence of spatial and environmental imprinting became even more evident by the finding of significant bacterial community differences between the four reef sites (Table 1). Remarkably, observed patterns not only reflected local site specificity (i.e. characteristic assemblages at different reefs), but also regional specificity (i.e. marked separation between both offshore *versus* each of the inshore reefs; Fig. S4). Although all reef sites share specific geological and hydrological features that are pivotal for local coral recruitment and proliferation (e.g. [1] and references therein), Røst, Trænadjupet, Tisler and Langenuen substantially differ in their geo- and hydrographical setting and structure. This seemed clearly reflected in the pronounced site specificity of water- and sediment-inhabiting bacteria. But also coral-generated microbial habitats exhibited reef-specific bacterial differences, more so in mucus than in branch samples, which basically confirmed the aforementioned differences from the local (meso-) to the regional (large-) scale. Those differences may involve reef-specific variations in mucus composition [53] as governed by environmental controls [54], or geographic fluctuations in coral reproduction strategy and genetic variability [45] as well as local coral food supply and quality [55], [56].

### Coral species and color

*L. pertusa* and *M. oculata* exhibited significantly different bacterial community structures, largely due to differences in OTU relative abundances. This corroborates evidence presented by Hansson *et al.* [29] who found DGGE signals from both species to group separately in NMDS and cluster analyses (yet, with >50% similarity). Like in our study, those corals originated from the same sampling location, where they occurred right next to each other, hence, a mere spatial separation of bacterial assemblages may not explain this pattern. Also, comparisons of 16S rRNA gene sequences from different studies [28]–[30] suggested divergence between *L. pertusa* and *M. oculata*-associated bacterial communities. As both coral species differ with respect to tissue-contained acid concentrations [57] as well as to the carbohydrate composition of their mucus [53], the involvement of host-related traits in structuring bacterial assemblages seems likely. Species-specific differences in mucus composition are also known from warm-water corals [58] and even held responsible for a close attuning of bacterial communities to host metabolism [59]. Host-related traits may also explain why *L. pertusa* and *M. oculata*-associated communities slightly varied in their response to spatial heterogeneity (Table S2A, C, B), indicating possible coral-specific differences in host-microbe interactions.

In contrast to the marked species-related patterns, no bacterial community differences were significantly linked to *L. pertusa* color type. This was due to the fact that none out of the 387–390 *L. pertusa*-associated OTUs were exclusively attributable to either white or red specimens. In contrast, Neulinger *et al.* [28] who previously studied bacterial associates of white and red *L. pertusa* from the Tautra Reef by 16S rRNA-based T-RFLP and sequence analysis, observed color-specific associations of distinct 16S rRNA gene phylotypes, but could not resolve community differences among color phenotypes by fingerprinting. In principle, ARISA offers more resolution than T-RFLP to detect OTU changes [60] as well as intra-genomic heterogeneities within closely related gene clusters [61]. In addition, the two studies differ with respect to sample origin and processing: While the Tautra corals were completely homogenized, introducing considerable amounts of tissue and carbonate skeleton into the analysis, the Røst corals were distinctly sampled for branch surface plaques and mucus exudates, targeting mainly interfacial communities on the coral surface. Hence, the consistent absence of significant phenotypic community change in both these fingerprinting studies may indeed indicate that bacterial associates of white and red *L. pertusa* are largely indiscriminative, at least by those techniques.

Noticeably, the finding of low numbers of shared bacterial types combined with significant community differences related to host taxonomy, are often interpreted as signs of host specificity, implying the selection of few beneficial associates as part of commensalistic or mutualistic relationships (e.g. [62] and references therein). Yet, host-microbe associations may only be called “specific” if the respective bacterial signatures are maintained over time and space, which is so far unresolved for bacteria associated with CWCs due to the lack of studies across spatial and temporal scales. Here, habitat-specific community patterns were conserved over all sites, including differences between low and high numbers of unique OTUs in coral-derived *versus* ambient microbial habitats, respectively. The divergence between bacterial communities associated with *L. pertusa* and *M. oculata* also appeared consistent, at least within a highly heterogeneous reef site such as Røst. Further, 3 out of 8 OTUs unique to *L. pertusa* branch at Røst-in also occurred in the branch samples at all other sites (i.e. Røst-out, Trænadjupet, Tisler, Langenuen), potentially indicating specialized bacterial associates. By contrast, such property was not identified for any of the mucus-contained OTUs, suggesting variable colonization of the mucus matrix by locally occurring communities. Most of the coral-associated OTUs were also found in the proximal sediments, suggesting a potential source for coral-associated bacteria. This was corroborated by the change of coral-associated bacterial communities from local (small- and meso-) to regional (large-) scale, resulting in biogeographic patterns comparable to those of ambient bacterial communities. Host specificity of bacterial types and communities should therefore be further investigated by colonization experiments with artificial surfaces.

### Bacterial biodiversity hotspots?

By providing a high degree of structural complexity and habitat heterogeneity, CWC reef ecosystems locally promote faunal diversity in the deep sea [7], [12]. The potential of CWC reefs to also represent biodiversity hotspots for bacterial communities seems, however, rather questionable, given the low bacterial OTU number (as proxy for bacterial richness) and the limited OTU specificity (as indicator of conserved bacterial signatures) associated with coral-generated surfaces detected in this study. Nevertheless, CWCs contributed 5% (OTUs found exclusively on corals) to 44% (OTUs shared between corals and water/sediments) of all bacterial OTUs detected at a single proliferating reef center (Røst-in). Furthermore, coral-generated surfaces, such as branch and mucus, were characterized by high bacterial community variation. Although high variability does not automatically translate into high local diversity, these small-scale differences may, combined with the meso- and large-scale community changes occurring within and between reef sites, increase bacterial community variation (beta diversity) in CWC ecosystems and contribute to specific source-sink dynamics in bacterial dispersal. In terms of bacterial types, however, it may rather be the reef sediments, water column or filtering invertebrates such as sponges, that contribute more to the overall bacterial diversity in CWC reefs than surfaces generated by *L. pertusa* or *M. oculata*. Those microbial habitats can feature high levels of organic matter and nutrients ([1] and references therein; [63]) and are usually characterized by diverse bacterial communities (e.g. [64], [65]).

Given that ARISA is based on the discrimination of ITS length among bacterial types, the technique cannot be used to precisely delineate the taxonomic levels at which the observed community changes operate [61]. Yet, based on our evaluation of community shifts at multiple levels of observation, future investigations may involve sequencing of ribosomal genes on representative samples to explore finer taxa-environment relationships. As reported by previous studies (e.g. [61], [66], [67]), bacterial community patterns derived from ARISA and sequencing (both Sanger and high-throughput) may, after all, be highly comparable and lead to very similar ecological conclusions.

### Conclusion

This study presents the first multi-scale survey of bacterial communities associated with *L. pertusa* and *M. oculata* in different CWC reefs, spanning a total of five study levels: (i) Microbial habitat type and (ii) coral species and color, as well as the spatial components (iii) geomorphologic reef zoning, (iv) reef boundary, and (v) reef location. Our findings revealed fundamental differences in bacterial specificity and community structure between distinct coral-generated (branch surface, mucus) and ambient microbial habitats (seawater, sediments), which appeared pivotal for determining bacterial variation over all observational levels investigated. Especially the high community variability associated with coral-derived surfaces represented a consistent feature of all four CWC reef sites under study. In addition, bacterial communities changed markedly from local (small- and meso-) to regional (large-) scale, suggesting significant biogeographic imprinting of seawater-, sediment- and even coral-associated communities, but weak microbe-host specificity. Overall, the relative effects of the different test parameters did not reflect any linear or even hierarchical relationship of bacterial community organization, but the deterministic effect of microbial habitat type and the strong effect of reef location seemed to play dominant roles in structuring bacteria in CWC reefs. The bacterial communities were structured across different spatial scales, from local within-reef habitats to regional across-reef systems, which may reflect the combined effects of local community history (e.g., community assembly and interactions) and environmental filtering. As exploring bacterial diversity in CWC reefs is but one of the first steps to a better understanding of coral-microbe relationships in complex deep-sea environments, further studies need to address how changes in bacterial (and general microbial) diversity affect the dynamics and functioning of CWC reef ecosystems – especially in the context of global environmental changes and the protection of CWC reefs as biodiversity hotspots.

## Materials and Methods

### Study sites

The four Norwegian CWC reef ecosystems sampled (Fig. 1A) comprised two offshore sites on the northern mid-Norwegian continental shelf (Røst, Trænadjupet), as well as two inshore sites located in the Norwegian Skagerrak (Tisler) and on the Norwegian South-West coast (Langenuen). A more detailed description, including sampling times, sampling coordinates, water depths and sample type (i.e. coral, seawater and sediment) yields, is provided as Supporting Information (Text S1; Fig. S1, Table S1).

### Hierarchical sampling design

Sampling was performed hierarchically, encompassing five levels of observation, including three spatial scales (Fig. 2). The first study level (HABITAT) comprised four potentially distinct types of microbial habitats associated with and surrounding a scleractinian coral colony in its reef environment: Coral branch surface, coral mucus, ambient seawater, and proximal sediments. The second level (COLOR, SPECIES) featured specific coral species (*L. pertusa*, *M. oculata*; Fig. 1B) and coral color types (white and red individuals of *L. pertusa*). Geomorphologic reef zoning, as prevailing at Røst, determined the third level (ZONE), including the terrace-covered ridge crest, the rubble- and sponge-dominated ridge slope, and the clay-bearing, sparsely populated inter-ridge depression in the reef center. At the fourth level (IN-OUT), the up-slope reef center (Røst-in) was compared with the down-slope reef periphery (Røst-out) in distances of 1 m, 10 m and 100 m away from the reef margin. The fifth level (REEF) allowed a comparison of the offshore Røst site with the nearby Trænadjupet site and the two inshore sites, Tisler and Langenuen. Arranged in a nested layout, each of these study levels was considered as integral part of the respective next higher level, along an increasing gradient of complexity ranging from level one (HABITAT) to level five (REEF).

At Røst, the main study site, where sampling focused on intra-reef differences (HABITAT, SPECIES/COLOR, ZONE, IN-OUT; Fig. 2), the collection of corals (*L. pertusa*, *M. oculata*), seawater and surface sediments was performed during two manned submersible dives down-slope across the reef (Fig. S1, Table S1). The first dive (ship station: PS 70/17-1) traversed two of the uppermost ridges in the reef center, while the second dive (ship station: PS 70/31-1) extended further down-slope to the reef periphery at a distance of approx. 2.5 km. At the other study sites (inter-reef differences: REEF; Fig. 2), sampling involved the collection of *L. pertusa*, seawater and sediments at random locations within the respective main reef area (Table S1).

### *In-situ* sample collection

Specimens of living CWCs were sampled by manned submersible (Røst, Trænadjupet) or video-assisted remotely operated vehicle (Tisler, Langenuen; Table S1). After visual assessment of each target colony *in situ*, one healthy looking fragment was picked from the colony's living outer rind using the manipulator arm, and placed into a separate compartment of the sampling reservoir. Onboard, each specimen was inspected for epigrowth, impurities or degeneration, before selecting an intact fragment (5–15 cm in length) for sub-sampling of coral-associated microbial habitats. Fragments needed for branch surface and mucus sampling were maintained in flow-through tanks with *in-situ* water at a temperature of 10–11°C for ≤30 min until subsequent processing. Seawater was sampled with 2l-Niskin bottles attached to the submersible (Røst, Trænadjupet), or mounted on a conductivity-temperature-depth rosette sampler (Langenuen) or video-assisted steel cable (Tisler; Table S1). Immediately after retrieval, 2l-water samples were kept at 4°C, filtered in 500 ml aliquots onto sterile polycarbonate membranes (0.22 µm pore size, Millipore, Billerica, MA), and stored at −20°C until further treatment. Surface sediments (approximately 0–5 cm sediment depth) were collected by custom sampling scoops operated via the submersible and vehicle manipulator arm (Røst, Trænadjupet, Tisler) or by Van-Veen grab (Langenuen; Table S1). Upon retrieval, sediment samples were immediately transferred into sterile 50-ml vials and stored at −20°C until further processing. At Røst, sediment sampling was not possible on the ridge crests owing to the density of the prevailing coral framework cover.

### Coral sub-sampling procedures

After gentle rinsing with sterile-filtered (Whatman, Maidstone, UK) local seawater, branch surfaces of living corals were sampled by scraping an area of up to 5 cm^2^ per fragment with sterile scalpel blades, yielding a mixture of surface plaques, coenosarc tissue, and calcareous particles. Scraping was carried out on the primary, and partly secondary, branches of each fragment, avoiding fragile outer branches as well as polyp calices. All material accumulated per fragment was directly transferred into a DNA extraction tube (see below). Freshly produced coral mucus was sampled by gently rinsing living coral fragments with sterile-filtered seawater and inducing mucus exudation through 2–5 min air exposure. After discarding exudate released during the first minute, subsequent production of up to 0.5 ml per fragment was collected directly from polyp surfaces by using sterile syringes. Resulting mucus-seawater mixture was concentrated onto sterile polycarbonate filters (Whatman), and frozen at −20°C until DNA extraction.

### DNA extraction

Total community DNA was extracted and purified with the Ultra Clean Soil DNA Kit (MoBio, Carlsbad, CA, USA) following the manufacturer's instructions for maximum yield, with slight modifications. Branch samples (scrapings from up to 5 cm^2^ surface per fragment) and sediment samples (3×1 g per scoop) were directly transferred into extraction tubes, mucus samples (up to 0.5 ml per fragment) and water samples (2–4×500 ml per Niskin bottle) on respective filter membranes. DNA yields were quantified by NanoDrop spectrophotometry (NanoDrop, Wilmington, DE). For a complete overview of sample units and replicates subjected to DNA extraction and subsequent community analyses, see Table S1.

### Community fingerprinting and multivariate analyses

Universal bacterial ARISA [68] based on 3 PCR replicates per sample, and subsequent binning into OTUs were carried out as described previously [69]. Based on a threshold of ≥0.09% in relative fluorescence intensity (individual peak areas divided by the total peak area of the respective sample) and 50 in fluorescence units, only ARISA fragments in the size range of 100–1000 bp were subjected to the binning procedure with a window size of 2 bp. Total numbers of ARISA-based OTUs (i.e. the number of bacterial types contained in each ARISA sample; used as relative proxy for richness), were assessed for mean difference by applying the non-parametric, omnibus Kruskal-Wallis test (KW), followed by pairwise Wilcoxon-Mann-Whitney tests (WMW). Multivariate patterns in community structure were analyzed based on Bray-Curtis dissimilarity matrices which were visually inspected by Non-metric MultiDimensional Scaling (NMDS), cluster analysis, and heat-mapping. Differences in community overlap between *a posteriori* defined sample categories were tested by Analysis of Similarity (ANOSIM) and corrected for multiple tests according to the Bonferroni criterion. Sources of bacterial community differences were further assessed by Permutational Multivariate Analysis of Variance (PERMANOVA; [70]) at three levels: (i) regional, inter-reef differences between the four different reef sites (REEF, HABITAT; Fig. 2) (ii) local, intra-reef differences in the whole Røst area (IN-OUT; Fig. 2), and (iii) local, intra-reef differences (ZONE, SPECIES, COLOR, HABITAT; Fig. 2) at the reef center of Røst. Numerical analyses were implemented in PAST v2.0 (Palaeontological Statistics) and in R v.2.9 (The R Project for Statistical Computing) using the standard and *vegan* packages, as well as custom scripts.

## Supporting Information

Text S1 **More detailed description of the study sites**.
(DOC)

Figure S1 **Geographical and topographical setting of sampling events at Røst.** (**A**) Røst bathymetry, including dive transects at Røst-in (reef center) and Røst-out (reef periphery; map: courtesy of V. Unnithan, JUB), (**B**) Røst transversal scheme (not to scale) indicating topographical reef structure, geomorphological reef zoning and single sampling stations (reef center: val = valley, slo = slope, cre = crest; reef periphery: 1/10/100 m = 1/10/100 m beyond the apparent reef margin).
(TIF)

Figure S2 **Partitioning of bacterial OTUs between distinct coral-associated and ambient microbial habitats.** Numbers indicate the amount of OTUs unique to each microbial habitat, or common to any two or all microbial habitats: (**A**) Bacterial OTUs associated with samples of white *L. pertusa (left)*, *red L. pertusa (middle)* or *M. oculata (right)* and their ambient environment at Røst-in, (**B**) Bacterial OTUs associated with samples of all coral species/colors and their ambient environment at Røst-in (*left*) or at all sites combined (*right*).
(TIF)

Figure S3 **Pairwise Bray-Curtis dissimilarity relationships between all community profiles.** Samples are grouped according to microbial habitat type, coral species and color, geomorphologic reef zoning, reef boundary (incl. distances away from the apparent reef margin), and reef site. Cell position corresponds to the symmetrical pairing of single sample groups. Cell shading indicates the magnitude of dissimilarity between sample pairs.
(TIF)

Figure S4 **NMDS ordinations of ARISA community profiles per microbial habitat.** For each microbial habitat type, differences in bacterial community structure are plotted as related to reef site, reef boundary, geomorphologic reef zoning, coral species and color. Objects represent consensus signals for all PCR triplicates per sample and share a more similar community structure when plotting closer to each other (Bray-Curtis distance). Stress values indicate the goodness-of-fit of the 2-dimensional representation compared to the original multi-dimensional matrix.
(TIF)

Table S1 **Overview on sampling events of living coral specimens, seawater and sediments at the different study sites.** wL = white *L. pertusa*, rL = red *L. pertusa*, rM = red *M. oculata*. ^a^Sample lost during processing. The full station list of ARKXXII/1a is available via the PANGAEA database at: http://www.pangaea.de/ddi?retr=events/HERMES/ARK-XXII_1a.retr&conf=events/CruiseReportHTML.conf&title=StationlistofcruiseARK-XXII/1a&format=html.
(DOC)

Table S2 **PERMANOVA of coral-associated bacterial variation done at Røst. A) Analyses considering A) reef boundary, i.e. in/out-reef location (IN-OUT) and B) geomorphologic reef zoning (ZONE) within Røst-in.** ^a^Source of variation. ^b^Amount of explained variation. ^c^Significance level, assessed by 999 random permutations (*** P≤0.001, ** P≤0.01, * P≤0.05). ^d^Significance level below Bonferroni correction threshold.
(DOC)
