# Supplementary material for: Spatial Scales of Bacterial Diversity in Cold-Water Coral Reef Ecosystems
Source: PLoS One. 2012 Mar 5;7(3):e32093. doi: 10.1371/journal.pone.0032093 (PMC3293894; doi:10.1371/journal.pone.0032093)
Supplement: Text S1 — More detailed description of the study sites. (DOC) [file pone.0032093.s001.doc]

**Study sites**

The Røst Reef (Fig. 1A), discovered in 2002, is regarded as the largest proliferating cold-water coral reef ecosystem in the world [1,2]. Situated on the northern mid-Norwegian continental slope at a water depth of 300–400 m, it forms a 35–50 km long and 3 km wide belt [3] covering parallel ridges in the headwall zone of the Trænadjupet submarine landslide [4–6]. The steep and rugged ridges of glacigenic origin rise several tens of meters above the surrounding seafloor, and decrease in spacing and size downslope [5,6]. The hydrodynamic regime around these ridges is controlled by the Norwegian Current entering the area from the south, resulting in strong northeast-oriented currents flowing approx. parallel to the reef [7,8]. Owing to the specific ridge morphology, current dynamics and resulting strong variation in post-slide hemipelagic sediment deposition [7], the Røst Reef complex features a distinct geomorphologic habitat zoning. Ridge crests and upper slope parts, consisting of hard glacial clay, are covered by a dense framework of living coral colonies which form giant apron-like terraces facing up-slope [3,9]. Main spatial contributors comprise the constructional Scleractinian species *L. pertusa* and *M. oculata*, which occur in several color types, mostly white and red (Fig. 1B). The lower slopes, characterized by coral rubble-bearing facies [9], are highly sponge-dominated, and coral occurrence is reduced to isolated living colonies originating from an occasional framework rupture at the ridge top and subsequent debris transport down-slope. The depressions between ridges comprise a fine-grained, clay to silt-bearing matrix with embedded dead coral fractions [9], and are populated by various sponge communities, with only few living colonies in between. In general, the highest degree of coral proliferation and density (reef center) is found up-slope, in immediate vicinity of the headwall (high-relief area; [3,1], JAGO team, pers. comment), while the down-slope reef periphery (low-relief area) exhibits only randomly occurring isolated colonies.

The Trænadjupet Reef (Fig. 1A) covers a circular embayment on the edge of Trænadjupet [10], an elongated transverse cross-shelf trough incising the mid-Norwegian shelf [11]. At 300–330 m water depth, it covers deltaic sandy fan deposits forming distinct cigar-shaped structures [12], and is exposed to the cyclonic circulation predominating in the Lofoten basin [8]. In absence of a distinct habitat zoning, most of the cigar-shaped elevations are covered by a fine-grained matrix of silt to clay and biogenic debris, with coral rubble and dead framework atop [9]. Living coral colonies of white *L. pertusa* are only found on some of the eastern tips of these structures, while sponges mark the dominant fraction of the overall reef community.

The Tisler Reef (Fig. 1A), first discovered and documented in 2002, represents one of the largest and shallowest coastal reefs known worldwide [13,14]. Situated on a sill in the Hvaler/Kosterfjord region, northeast of the Tisler islands in the Norwegian Skagerrak, it encompasses an area of about 1200 m × 200 m at a water depth of 70–160 m [13,14]. Due to the Kosterfjord deep-water connection to the open Skagerrak, the reef is exposed to strong currents being forced through a long, deep gully in northwest-southeast direction [15]. The live coral cover is dominated by many large colonies of *L. pertusa*, which occur in several color types and reach sizes of up to 2 m in diameter [13]. Also sponges constitute an integral part of the reef structure [16]. In the distal reef areas, large dead coral structures indicate severe trawl damage and suggest the original size of the living Tisler reef as about twice its present size [15]; T. Lundälv, pers. comment).

The Langenuen Fjord in West-Norway near Bergen (Fig. 1A), is a north-south water passage connecting the Hardangerfjord with the Korsfjord. One of the several patch reefs covering the rocky, steep slopes of the northern fjord section is located near Landrøyodden at a water depth of approximately 80–420 m. On top of rock- and rubble-bearing facies, living colonies of *L. pertus*a and *M. oculata* occur among different types of sponges and other sessile hard-bottom invertebrates.

**References**

1. Nordgulen O, Bargel TH, Longva O, Ottesen D (2006) A preliminary study of Lofoten as a potential World Heritage Site based on natural criteria. Geol Survey Norway.

2. Thorsnes T, Fosså JH, Christensen O (2004) Deep-water coral reefs. Acoustic recognition and geological setting. Hydro International 8: 26–29.

3. Fosså JH, Lindberg B, Christensen O, Lundälv T, Svällingen I, et al. (2005) Mapping of Lophelia reefs in Norway: experiences and survey methods. In: Freiwald A, Roberts JM, editors. Cold-water corals and ecosystems*.* Heidelberg, Germany. pp. 359–391.

4. Damuth JE (1978) Echo character of Norwegian-Greenland Sea: relationship to Quarternary sedimentation. Mar Geol 28: 1–36.

5. Laberg JS, Vorren TO (2000) The Trænadjupet Slide, offshore Norway – morphology, evacuation and triggering mechanisms. Mar Geol 171: 95–114.

6. Laberg JS, Vorren TO, Mienert J, Evans D, Lindberg B, et al. (2002) Late Quarternary pleoenvironment and chronology in the Trænadjupet Slide area offshore Norway. Mar Geol 188: 35–60.

7. Laberg JS, Vorren TO, Mienert J, Bryn P, Lien R (2002) The Trænadjupet Slide: a large slope failure affecting the continental margin off Norway 4,000 years ago. Geo Mar Lett 22: 19–24.

8. Poulain PM, Warn-Varnas A, Niiler PP (1996) Near-surface circulation of the Nordic seas as measured by Lagrangian drifters*.* J Geophys Res Oceans 101: 18237–18258.

9. Wehrmann LM, Knab NJ, Pirlet H, Unnithan V, Wild C, et al. (2009) Carbon mineralization and carbonate preservation in modern cold-water coral reef sediments on the Norwegian shelf. Biogeosci 6: 663–680.

10. Hovland M, Mortensen PB (1999) Norske korallrev og prosesser i havbunnen. Bergen: John Grieg.

11. Ottesen D, Rise L, Knies J, Olsen L, Henriksen S (2005) The Vestfjorden-Trænadjupet paleo-ice stream drainage system, mid-Norwegian continental shelf. Mar Geol 218: 175–189.

12. Hovland M, Ottesen D, Thorsnes T, Fosså JH, Bryn P (2005) Occurrence and implications of large Lophelia-reefs offshore mid-Norway. In: Wandas B, Nystuen JP, Eide E, Gradstein G, editors. Onshore-offshore relationships on the North Atlantic margin. Norwegian Petrolium Society (NPF) Special Publications. Amsterdam: Elsevier. pp. 265-270.

13. Jonsson LG (2006) Ecology of three coastal cold-water cnidarians, in particular the sclaractininan *Lophelia pertusa.* PhD thesis. Göteborg: Göteborg University.

14. Lundälv T (2004) Kartläggning av biologiska värden i djupare delar av Yttre Hvaler, nordöstra Skagerrak, med ROV-teknik. Report Østfold fylke, pp 1–34.

15. Lavaleye M, Duineveld G, Lundälv T, White M, Guihen D, et al. (2009) Cold-water corals on the Tisler Reef: Preliminary observations on the dynamic reef environment. Oceanogr 22: 55–62.

16. Purser A, Bergmann M, Lundälv T, Ontrup J, Nattkemper TW (2009) Use of machine-learn algorithms for the automated detection of cold-water coral habitats. Mar Ecol Prog Ser 397: 241–251.
