# Supplementary figures and images for: Spatial Scales of Bacterial Diversity in Cold-Water Coral Reef Ecosystems
Source: PLoS One. 2012 Mar 5;7(3):e32093. doi: 10.1371/journal.pone.0032093 (PMC3293894; doi:10.1371/journal.pone.0032093)

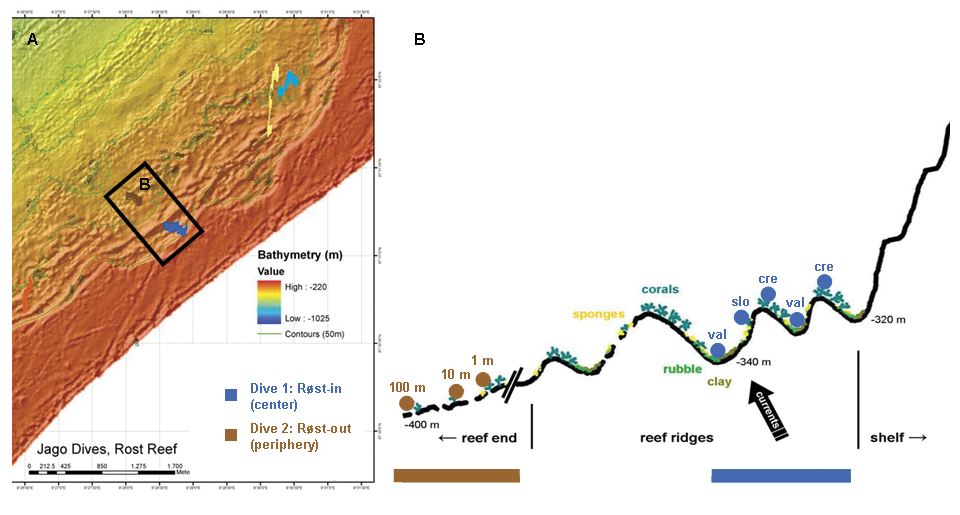

Supplement: Figure S1 — Geographical and topographical setting of sampling events at Røst. (A) Røst bathymetry, including dive transects at Røst-in (reef center) and Røst-out (reef periphery; map: courtesy of V. Unnithan, JUB), (B) Røst transversal scheme (not to scale) indicating topographical reef structure, geomorphological reef zoning and single sampling stations (reef center: val = valley, slo = slope, cre = crest; reef periphery: 1/10/100 m = 1/10/100 m beyond the apparent reef margin). (TIF) [file pone.0032093.s002.tif]

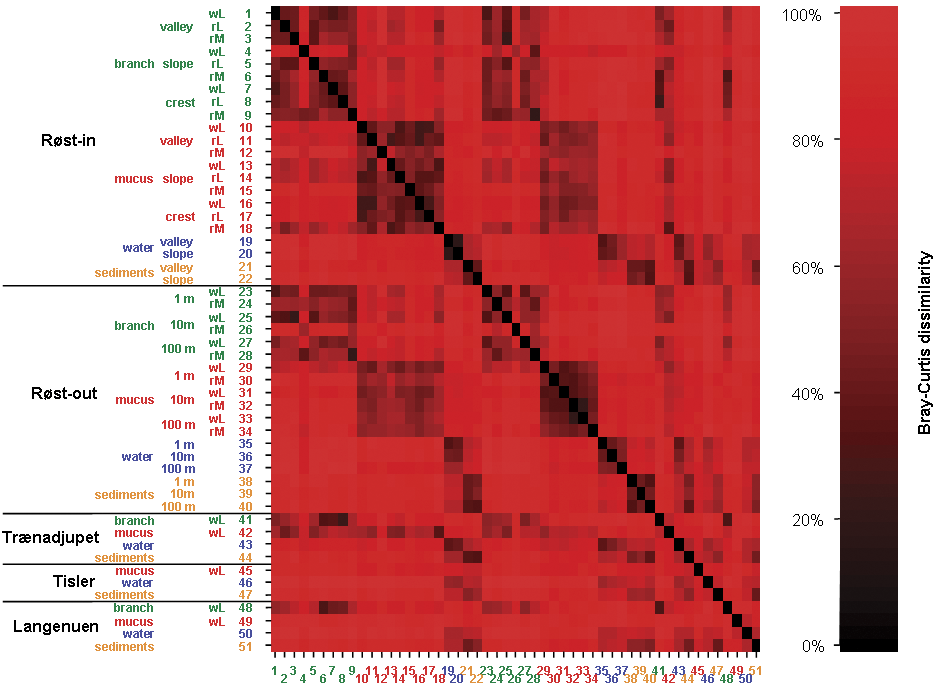

Supplement: Figure S2 — Partitioning of bacterial OTUs between distinct coral-associated and ambient microbial habitats. Numbers indicate the amount of OTUs unique to each microbial habitat, or common to any two or all microbial habitats: (A) Bacterial OTUs associated with samples of white L. pertusa (left), red L. pertusa (middle) or M. oculata (right) and their ambient environment at Røst-in, (B) Bacterial OTUs associated with samples of all coral species/colors and their ambient environment at Røst-in (left) or at all sites combined (right). (TIF) [file pone.0032093.s003.tif]

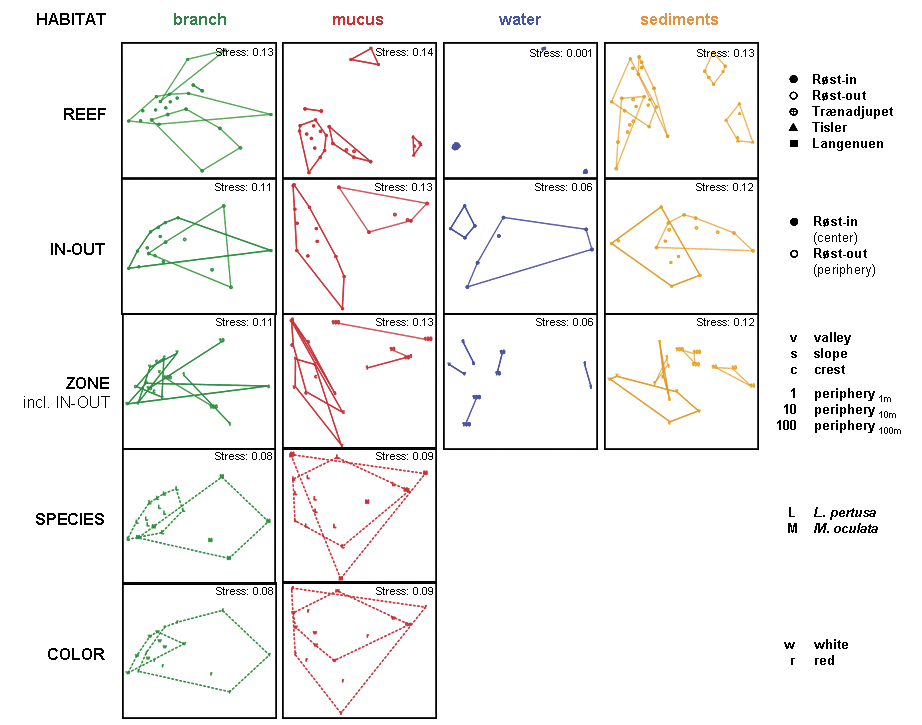

Supplement: Figure S4 — NMDS ordinations of ARISA community profiles per microbial habitat. For each microbial habitat type, differences in bacterial community structure are plotted as related to reef site, reef boundary, geomorphologic reef zoning, coral species and color. Objects represent consensus signals for all PCR triplicates per sample and share a more similar community structure when plotting closer to each other (Bray-Curtis distance). Stress values indicate the goodness-of-fit of the 2-dimensional representation compared to the original multi-dimensional matrix. (TIF) [file pone.0032093.s005.tif]
