# Supplementary material for: Spatial Scales of Bacterial Diversity in Cold-Water Coral Reef Ecosystems
Source: PLoS One. 2012 Mar 5;7(3):e32093. doi: 10.1371/journal.pone.0032093 (PMC3293894; doi:10.1371/journal.pone.0032093)
Supplement: Table S1 — Overview on sampling events of living coral specimens, seawater and sediments at the different study sites. wL = white L. pertusa, rL = red L. pertusa, rM = red M. oculata. aSample lost during processing. The full station list of ARKXXII/1a is available via the PANGAEA database at: http://www.pangaea.de/ddi?retr=events/HERMES/ARK-XXII_1a.retr&conf=events/CruiseReportHTML.conf&title=StationlistofcruiseARK-XXII/1a&format=html. (DOC) [file pone.0032093.s006.doc]

| **Table S1. Overview on sampling events of living coral specimens, seawater, and sediments at the different study sites.** | | | | | | | | | | | |
| --- | --- | --- | --- | --- | --- | --- | --- | --- | --- | --- | --- |
| **Study**  **site** | **Research vessel** | **Expedition**  **ID** | **Event ID**  **and date** | **Sampling device** | **Sampling position** | **Water**  **depth** | **Reef**  **zone** | **Sample**  **type** | **Sample amount** | **Coral**  **type** | **Units used**  **for ARISA** |
| **Røst-in** | *RV Polarstern* | ARK XXXII/1a | PS 70/17-1  10 Jun, 2007 | submersible *JAGO* | 67°31’09’’ N  09°28’47’’ E | 320 m | ridge  crest | coral | 3 colonies | wL, rL, rM | 3x 1 fragment |
|  |  |  |  |  | 67°31’09’’ N  09°28’47’’ E | 326 m | ridge  valley | coral  sediments | 2 colonies  50 ml | wL, rL  – | 2x 1 fragment  3x 1 g |
|  |  |  |  |  | 67°31’10’’ N  09°28’42’’ E | 318 m | ridge  (crest) ^a^ | coral  (water) ^a^ | 2 colonies  (2 l) ^a^ | wL, rL  – | 2x 1 fragment  – ^a^ |
|  |  |  |  |  | 67°31’10’’ N  09°28’42’’ E | 328 m | ridge  slope | coral  water  sediments | 3 colonies  2 l  50 ml | wL, rL, rM  –  – | 3x 1 fragment  2x 1l  3x 1 g |
|  |  |  |  |  | 67°31’10’’ N  09°28’35’’ E | 340 m | ridge  valley | coral  water  sediment | 2 colonies  2 l  50 ml | wL, rM  –  – | 2x 1 fragment  2x 1l  3x 1 g |
| **Røst-out** | *RV Polarstern* | ARK XXXII/1a | PS 70/31-1  15 Jun, 2007 | submersible *JAGO* | 67°31’18’’ N  09°28’12’’ E | 365 m | 1 m  distance | coral  water  sediments | 2 colonies  1 l  50 ml | wL, rM  –  – | 2x 1 fragment  1x 1l  3x 1 g |
|  |  |  |  |  | 67°31’19’’ N  09°28’11’’ E | 370 m | 10 m  distance | coral  water  sediments | 2 colonies  2 l  50 ml | wL, rM  –  – | 2x 1 fragment  2x 1 l  3x 1 g |
|  |  |  |  |  | 67°31’20’’ N  09°28’01’’ E | 388 m | 100 m  distance | coral  water  sediments | 2 colonies  2 l  50 ml | wL, rM  –  – | 2x 1 fragment  2x 1l  3x 1 g |
| **Trænadjupet** | *RV Polarstern* | ARK XXXII/1a | PS 70/27-1  14 Jun, 2007 | submersible *JAGO* | 66°58’21’’ N  11°06’37’’ E | 302 m | – | coral  water  sediments | 1 colony  2 l  50 ml | wL  –  – | 1x 1 fragment  2x 1 l  3x 1 g |
|  |  |  |  |  | 66°58’22’’ N  11°06’33’’ E | 299 m | – | coral  sediments | 1 colony  50 ml | wL | 1x 1 fragment  3x 1 g |
| **Tisler** | *RV Lophelia* | – | –  21 May, 2008 | ROV *SubFighter* | 58°59’51’’ N  10°57’34’’ E | 107 m | – | coral | 2 colonies | wL | 2x 1 fragment |
|  |  |  |  |  | 58°59’53’’ N  10°57’40’’ E | 91 m | – | coral | 2 colonies | wL | 2x 1 fragment |
|  |  |  |  |  | 58°59’50’’ N  10°57’39’’ E | 100 m | – | coral | 1 colony | wL | 1x 1 fragment |
|  |  |  |  |  | 58°59’39’’ N  10°57’50’’ E | 105 m | – | water  sediments | 2 l  50 ml | –  – | 2x 1l  3x 1g |
|  |  |  |  |  | 58°59’51’’ N  10°57’39’’ E | 97 m | – | water  sediments | 2 l  50 ml | –  – | 2x 1l  3x 1g |
| **Langenuen** | *RV G.O. Sars* | 2006118 | –  07 Dec, 2006 | ROV  *Aglantha* | 59°58’30’’ N  05°22’30’’ E | 259 m | – | coral | 1 colony | wL | 1x 3 fragments |
|  | *RV G.O. Sars* | 2006118 | –  07 Dec, 2006 | CTD | – | 250 m | – | water | 4 l | – | 2x 1l |
|  | *RV H. Brattstrøm* | – | –  09 Oct, 2006 | VanVeen  grab | 59°56’30’’ N  05°28’30’’ E | 175 m | – | sediments | 50 ml | – | 6x 1g |
| Abbreviations: wL = white *L. pertusa*, rL =red *L. pertusa*, rM = red *M. oculata*. ^a^ Sample lost during processing. The full station list of ARKXXII/1a is available via the PANGAEA database at:  <http://www.pangaea.de/ddi?retr=events/HERMES/ARK-XXII_1a.retr&conf=events/CruiseReportHTML.conf&title=Station+list+of+cruise+ARK-XXII/1a&format=html> | | | | | | | | | | | |
