# Supplementary material for: Spatial Scales of Bacterial Diversity in Cold-Water Coral Reef Ecosystems
Source: PLoS One. 2012 Mar 5;7(3):e32093. doi: 10.1371/journal.pone.0032093 (PMC3293894; doi:10.1371/journal.pone.0032093)
Supplement: Table S2 — PERMANOVA of coral-associated bacterial variation done at Røst. A) Analyses considering A) reef boundary, i.e. in/out-reef location (IN-OUT) and B) geomorphologic reef zoning (ZONE) within Røst-in. aSource of variation. bAmount of explained variation. cSignificance level, assessed by 999 random permutations (*** P≤0.001, ** P≤0.01, * P≤0.05). dSignificance level below Bonferroni correction threshold. (DOC) [file pone.0032093.s007.doc]

**Table S2A**

| **Test factor** ^a^ | **Test group** | | **R^2^**  ^b^ | **F-ratio** | **P ^c^** |
| --- | --- | --- | --- | --- | --- |
| IN-OUT | all *L. pertusa* samples | | 0.679 | 1.603 | 0.142 |
| IN-OUT | *L. pertusa* | branch | 0.105 | 1.167 | 0.294 |
| IN-OUT | *L. pertusa* | mucus | 0.326 | 4.828 | 0.001 ^*** d^ |
| IN-OUT | all *M. oculata* samples | | 0.129 | 1.481 | 0.153 |
| IN-OUT | *M. oculata* | branch | 0.269 | 1.475 | 0.001 ^*** d^ |
| IN-OUT | *M. oculata* | mucus | 0.326 | 3.002 | 0.036 ^*^ |

**Table S2B**

| **Test factor** ^a^ | | **Test group** | | **R^2^**  ^b^ | **F-ratio** | **P** ^c^ |
| --- | --- | --- | --- | --- | --- | --- |
| ZONE | | all *L. pertusa* samples | | 0.102 | 0.853 | 0.5 |
| ZONE | | *L. pertusa* | branch | 0.339 | 1.542 | 0.081 |
| ZONE | | *L. pertusa* | mucus | 0.313 | 1.368 | 0.139 |
| ZONE | all *M. oculata* samples | | | 0.277 | 0.574 | 0.677 |
| ZONE | *M. oculata* | | branch | 1.000 | 0.000 | 0.001 ^*** d^ |
| ZONE | *M. oculata* | | mucus | 1.000 | 0.000 | 0.001 ^*** d^ |
